# Chronic Hypercapnic Respiratory Failure in a Patient With Smith-Magenis Syndrome: A Case Report

**DOI:** 10.7759/cureus.72980

**Published:** 2024-11-04

**Authors:** Qasim Alhaj Ali, Ingyin May, Debarghya Chakraborty, Chia Ling Tey

**Affiliations:** 1 Acute Medicine, King's College Hospital NHS Foundation Trust, London, GBR; 2 General Medicine, King's College Hospital NHS Foundation Trust, London, GBR; 3 Respiratory Medicine, King's College Hospital NHS Foundation Trust, London, GBR

**Keywords:** hypercapneic respiratory failure, hypercapnia, obesity hypoventilation syndrome, obstructive sleep apnea, smith-magenis syndrome

## Abstract

Smith-Magenis syndrome (SMS) is a genetic disorder characterized by intellectual disability, behavioral challenges, and distinctive physical abnormalities. This case report describes a patient with SMS who presented with pneumonia and was found to have chronic hypercapnic respiratory failure, attributed to kyphoscoliosis and obesity-related conditions such as obesity hypoventilation syndrome and obstructive sleep apnea. Following treatment with non-invasive ventilation (NIV), the patient’s baseline oxygen levels improved, and she was discharged with domiciliary NIV and respiratory follow-up. This report highlights the susceptibility of SMS patients to respiratory complications and emphasizes the importance of a comprehensive approach to managing these challenges.

## Introduction

Smith-Magenis syndrome (SMS) is a genetic disorder caused by a microdeletion on chromosome 17p11.2, with a prevalence of 1 in 15,000 to 25,000 live births. It is characterized by intellectual disability, speech and motor delays, sleep disturbances, and a range of physical features, including a square-shaped face, short stature, obesity, and craniofacial anomalies. Behavioral issues such as self-injurious behavior and attention-seeking tendencies are also common among individuals with SMS [1].

Although the neurodevelopmental and behavioral aspects of SMS are well documented, the syndrome's impact on respiratory health is less frequently addressed. Patients with SMS are predisposed to various respiratory challenges due to their distinctive anatomical and functional features, which can lead to impaired ventilation and oxygenation, potentially resulting in respiratory failure [2-5].

## Case presentation

A 20-year-old female, diagnosed with SMS at the age of two, presented to the emergency department with a five-day history of vomiting and constipation. CT imaging of her abdomen ruled out bowel obstruction but showed fecal impaction, which was treated with an enema, and she was discharged after her bowels opened. However, the following day, her mother found her drowsy at home, with an oxygen saturation of 44%. Upon ambulance arrival, the patient had an oxygen saturation of 74% on room air and was promptly administered oxygen therapy.

She primarily complained of abdominal pain and denied symptoms such as cough, chest pain, or fever. Her mother reported a longstanding history of sleep disturbances and snoring, with the snoring worsening in the month leading up to this presentation. Additionally, the patient had been sleeping propped up on pillows for several months and had recently shown a noticeable decrease in mobility.

Socially, she lived with her family and was entirely dependent on them for her care. She is reported to be socially active at school and with friends.

On examination, her heart rate was 132 beats per minute, her blood pressure was 141/96 mmHg, and her temperature was 37.8°C. The patient was obese with a body mass index (BMI) of 41 and had visible kyphosis. Chest auscultation revealed reduced air entry bilaterally, with no wheezes or crackles. Cardiac examination was unremarkable with no murmurs, and her abdomen was soft but diffusely tender.

Investigations

Arterial blood gas analysis on 40% FiO_2_ showed compensated type 2 respiratory failure with the following results: pH of 7.409, pCO_2_ of 6.33 kPa, pO_2_ of 13 kPa, HCO_3_ of 29.9 mmol/L, base excess of 4.6 mmol/L (Table 1). Blood tests revealed normal white blood cell count (7.7 × 10⁹/L), elevated C-reactive protein (65 mg/L), significantly raised alanine aminotransferase (ALT) (1,923 U/L), and elevated N-terminal pro-b-type natriuretic peptide (NT-proBNP) (1,163 ng/L) (Table 2). The initial blood culture during admission grew coagulase-negative Staphylococcus (likely contaminant). Respiratory viral screening and atypical pneumonia screens were negative. Additionally, the electrocardiogram showed sinus rhythm at 85 beats per minute.

**Table 1:** Arterial blood gas obtained on 40% FiO2

Parameter	Result	Normal value
pH	7.409	7.350-7.450
pCO_2_	6.33 kPa	4.70-6.00 kPa
pO_2_	13 kPa	10.60-13.30 kPa
HCO_3_	29.9 mmol/L	22.0-28.0 mmol/L
Base excess	4.6 mmol/L	-2.0 to 2.0 mmol/L

**Table 2:** Laboratory values at the point of admission NT-proBNP: N-terminal pro b-type natriuretic peptide

Parameter	Result	Normal value
White blood cell count	7.7 × 10⁹/L	2.9-9.6 × 10⁹/L
Neutrophils	6.19 × 10⁹/L	1.50-6.10 × 10⁹/L
Lymphocytes	1.09 × 10⁹/L	0.80-3.50 × 10⁹/L
C-reactive protein	65 mg/L	<5 mg/L
NT-proBNP	1,163 ng/L	<400 ng/L
Alanine aminotransferase	1,923 U/L	10-35 U/L
Alkaline phosphatase	137 U/L	30-130 U/L
Total bilirubin	16 μmol/L	<21 μmol/L

**Figure 1:**
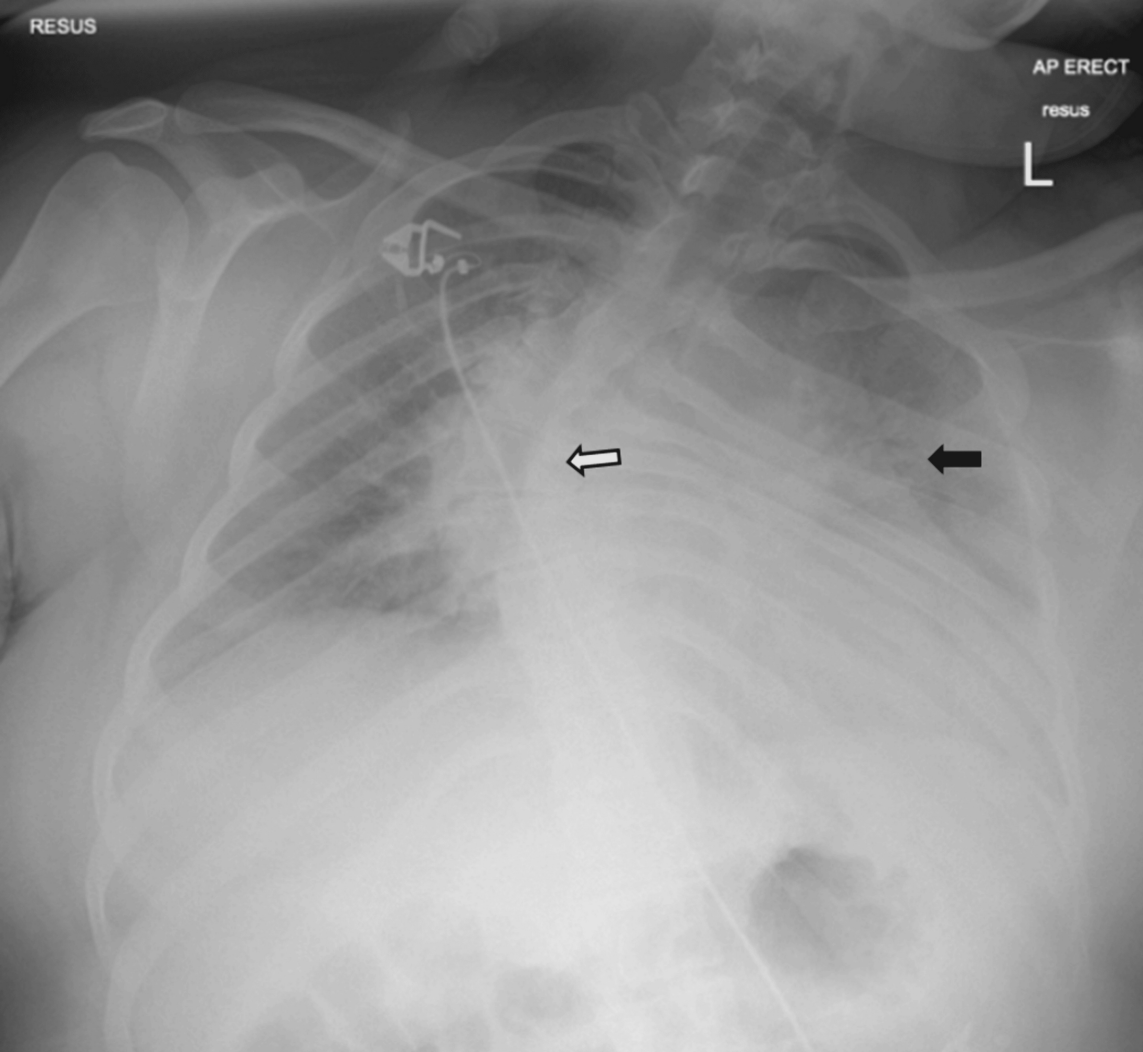
Chest X-ray shows hazy airspace opacities (black arrow) in both lung fields. Scoliosis of the thoracic spine is also noted (white arrow)

**Figure 2:**
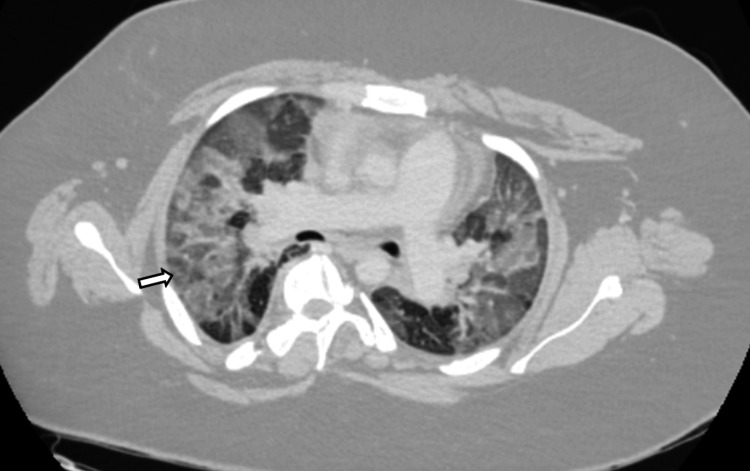
CT abdomen and pelvis study demonstrating signs of ground glass opacification in the lung fields (white arrow)

**Figure 3:**
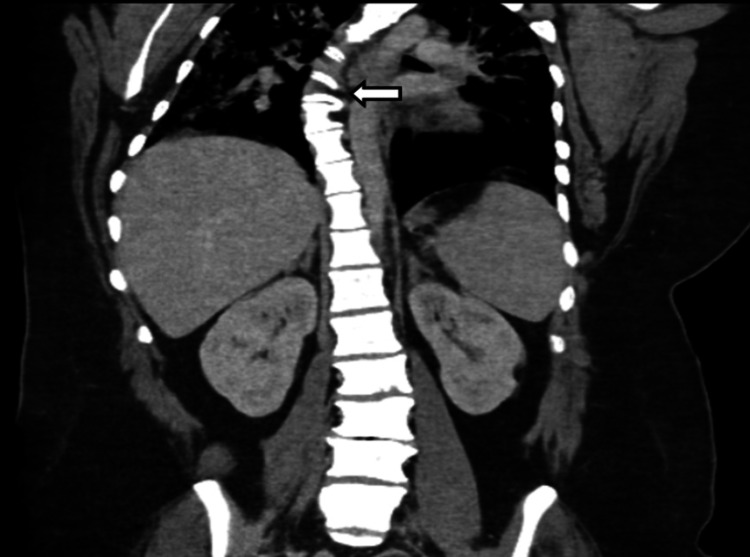
Coronal view of the CT abdomen and pelvis reveals marked scoliosis of the thoracic spine (white arrow)

Chest X-ray revealed bilateral hazy airspace opacities (Figure 1). CT imaging from the previous day showed bilateral ground glass infiltrates and mosaicism (Figure 2). Both imaging studies also revealed scoliosis (Figures 1, 3).

Overnight pulse oximetry, arranged later during admission to investigate possible obstructive sleep apnea (OSA) or obesity hypoventilation syndrome (OHS), confirmed severe nocturnal hypoxia, with an oxygen desaturation index (ODI) of 89.43 events per hour and a mean oxygen saturation of 75%.

Further tests were conducted, including a screening for viral and autoimmune hepatitis and a gallbladder ultrasound, both of which returned negative results.

Treatment

The patient initially received oxygen therapy at 15 L/min using a non-rebreather mask. This was later transitioned to targeted oxygen therapy at 8 L/min, aiming for oxygen saturation targets of 88-92% to avoid worsening hypercapnia. Although the bilateral ground-glass opacifications would typically indicate a viral infection, she was empirically treated for bacterial community-acquired pneumonia with antibiotics such as co-amoxiclav and doxycycline. Intravenous furosemide was briefly administered due to concerns of pulmonary edema. Laxatives were provided to relieve constipation.

With the diagnosis of OSA/OHS, nocturnal non-invasive ventilation (NIV) was initiated to support her nighttime breathing. The initial NIV settings were set at 12 cm H_2_O for inspiratory positive airway pressure (IPAP), and 4 cm H_2_O for expiratory positive airway pressure (EPAP), with gradual adjustments to 26 cm H_2_O for IPAP, and 8 cm H_2_O for EPAP.

Outcome

During the hospital stay, the patient’s oxygen therapy requirements steadily decreased, with low-flow oxygen therapy (0.5-1 L/min) needed for two weeks. She was able to maintain target oxygen saturation levels of 88-92% with the use of nocturnal NIV. A repeat chest X-ray showed improvement in the bilateral lung infiltrates, consistent with resolving pneumonia. Liver enzyme levels gradually decreased (ALT decreased from 1,923 to 154 U/L), and NT-proBNP levels returned to normal (Table 3). An echocardiogram was not performed, as heart failure was deemed unlikely.

**Table 3:** Laboratory values before discharge NT-proBNP: N-terminal pro b-type natriuretic peptide

Parameter	Result	Normal value
White blood cell count	5.9 × 10⁹/L	2.9-9.6 × 10⁹/L
Neutrophils	4.37 × 10⁹/L	1.50-6.10 × 10⁹/L
Lymphocytes	0.80 × 10⁹/L	0.80-3.50 × 10⁹/L
C-reactive protein	11 mg/L	<5 mg/L
NT-proBNP	<50 ng/L	<400 ng/L
Alanine aminotransferase	154 U/L	10-35 U/L
Alkaline phosphatase	125 U/L	30-130 U/L
Total bilirubin	4 μmol/L	<21 μmol/L

The patient was discharged with a home-based NIV, with a chest CT scheduled in six weeks. Further respiratory follow-up was planned for ongoing care, including long-term NIV management.

## Discussion

This patient’s chronic hypercapnic respiratory failure was likely multifactorial, with obesity playing a central role. Obesity, a common finding in SMS patients [1], often leads to respiratory issues such as reduced chest wall compliance, reduced lung volumes, increased airway resistance, and impaired diaphragmatic movement, resulting in hypoventilation and hypercapnia, key features of OHS [2]. The patient also exhibited signs of OSA, which further impairs respiratory function through repeated airway collapse and intermittent hypoxia [2]. Additionally, orofacial and laryngeal abnormalities frequently observed in SMS [1] have been shown to contribute to airway obstruction and worsening of OSA symptoms [3,4].

Scoliosis affects at least 30% of SMS patients [6,7]. In this case, the patient had kyphoscoliosis, a combination of scoliosis and kyphosis. Severe scoliosis leads to chest wall deformities that reduce compliance and limit lung expansion [5]. Kyphosis, which involves an exaggerated forward curvature of the spine, further compresses the chest wall and restricts diaphragm movement, leading to even more compromised ventilation [8].

Our patient presented acutely with pneumonia, which exacerbated the underlying respiratory failure. This highlights how such cases may involve complex clinical challenges that require rapid intervention and specialist support from a respiratory team.

The use of nocturnal NIV in patients with SMS is a crucial step in managing chronic respiratory failure. This intervention can improve hypercapnia, reduce daytime sleepiness, decrease hospital admissions, and may also improve some behavioral issues associated with SMS [9-11]. Ongoing follow-up with a specialized sleep and ventilation team is necessary to optimize NIV settings and address complex respiratory needs. A multidisciplinary team (MDT) approach, involving chest physiotherapists and respiratory nurses, provides patients and caregivers with essential training, maintenance, and support, contributing to better patient outcomes and an improved quality of life [12].

## Conclusions

In conclusion, chronic respiratory failure in SMS patients likely arises from a combination of factors and requires a comprehensive approach. NIV use may lead to significant improvements in outcomes and quality of life. Future research should investigate optimal screening and intervention strategies to mitigate respiratory complications in SMS.
